# Reply: Identification of isolated tumour cells by detection of CEA and CK mRNA in peritoneal lavage fluid of gastric cancer patients

**DOI:** 10.1038/sj.bjc.6604190

**Published:** 2008-01-15

**Authors:** K Katsuragi, M Yashiro, K Hirakawa

**Affiliations:** 1 Department of Surgical Oncology, Osaka City University Graduate School of Medicine, 1-4-3 Asahi-machi, Abeno-ku, Osaka 545-8585, Japan

**Sir**,

We thank Dr Kowalewska and his colleagues for their interest in our study (Katsuragi *et al*, 2007) and wish to respond to their comments. Kowalewska *et al* raised an important issue regarding the nonspecific expression of tumour markers by activated lymphoid cells. We are aware of the nonspecific expression of *CEA* and *CK20* by normal cells including haematopoietic cells or mesothelial cells in peritoneal fluid. Then, the peritoneal lavage specimens obtained from patients with cholecystolithiasis who had cholecystitis for cholecystectomy was used as the negative control. The cutoff value was determined based on the *CEA/GAPDH* mRNA ratio and *CK20/GAPDH* mRNA ratio levels in the peritoneal lavage fluid of the negative control and T1 samples. No sample from patients with cholecystolithiasis was over the cutoff value. Taken together, the standard curve of *CK20* and *CEA* mRNA using a gastric cancer cell line was drawn by dilution with normal monocytes at various ratios (Osaka *et al*, 2004). *CK20* and *CEA* mRNA were detectable by RT-PCR at a concentration as low as one cancer cell per 10^6^ lymphocytes. Although inflammatory cells express *CEA* and *CK20* mRNA, the expression level might be obviously different between cancer and inflammatory cells. Larger samples will be required in future studies to define the impact of this technology on prognosis and/or disease monitoring especially in patients with inflammatory conditions.

In this study, patients were followed up for less than 5 years with the median follow-up at analysis being 32 months. Although 35% of cytology-negative PCR-positive patients developed no peritoneal metastasis at this period, the rate of peritoneal recurrence in these patients might be increased in the future follow-up study. We will be continuing the follow-up of these patients for more than 5 years. The term ‘isolated tumour cells’ was used according to the definition of UICC (Sobin and Wittekind, 2002). Isolated tumour cells are single tumour cells or small clusters of cells that are usually detected by immunohistochemistry or molecular methods in peritoneal lavage fluid of isolated tumour cells from gastric cancer patients.
